# The Diagnosis of Granulomatosis With Polyangiitis When Serology and Biopsies are Negative

**DOI:** 10.1002/oto2.138

**Published:** 2024-05-15

**Authors:** Charles Teames, Julie Highland, Daniel Cox, Mark Elstad, Curry Koening, Marshall Smith

**Affiliations:** ^1^ Spencer Fox Eccles School of Medicine University of Utah Health Salt Lake City Utah USA; ^2^ Department of Otolaryngology University of Utah Health Salt Lake City Utah USA; ^3^ Department of Internal Medicine University of Utah Health Salt Lake City Utah USA; ^4^ Department of Internal Medicine University of Texas at Austin Austin Texas USA

**Keywords:** airway pathology, granulomatosis with polyangiitis, sinus pathology, vasculitis

## Abstract

**Objective:**

Granulomatosis with polyangiitis (GPA) is a potentially fatal condition which often manifests in the head and neck. Currently, diagnosis relies on antineutrophil cytoplasmic autoantibody (c‐ANCA) serology and mucosal or renal biopsy. However, a significant proportion of patients with GPA limited to the head and neck are seronegative and biopsy negative. This study evaluates the role of clinical diagnosis of GPA in the absence of positive laboratory findings.

**Study Design:**

Case series with chart review.

**Setting:**

Academic Tertiary Medical Center.

**Methods:**

This was a retrospective review of 143 patients treated in an outpatient otolaryngology clinic at a tertiary care hospital for known or suspected GPA from 1998 to 2021. Presenting symptoms, C‐ANCA status at initial presentation, biopsy results, long‐term serology results, and time to initiation of treatment were analyzed.

**Results:**

Twenty‐six of 143 (18.2%) patients were seronegative; only 3 of these patients (12%) had positive biopsies. Seventeen (73.9%) of these patients presented with nasal and sinus disease and 12 (52.2%) presented with airway involvement. Only 4 (17.4%) patients had renal involvement. Delay in treatment of patients with negative laboratory workup ranged from 0 months to 11 years. All patients who were seronegative and/or biopsy negative at presentation responded clinically to immunosuppressive therapy.

**Conclusion:**

GPA cases are often limited to the upper respiratory tract, making diagnosis difficult, particularly in seronegative patients. These results suggest that, when GPA is suspected, despite negative serology, the diagnosis of GPA should be made on clinical grounds, and empiric therapy encouraged to prevent delay in treatment.

Granulomatosis with polyangiitis (GPA, formerly Wegener's Granulomatosis) is a systemic disorder characterized by necrosing inflammation of the microvasculature of small vessels.^1^ The first series of patients were described in the 1930s,^2^ and the condition has since typically been associated with cytoplasmic‐staining antineutrophil cytoplasmic antibodies (c‐ANCAs) directed against proteinase 3 on serology.^3^ However, the positivity rate of c‐ANCA in serum from patients with GPA is only approximately 80%, leaving a substantial subset of seronegative patients who may receive a delayed or inaccurate diagnosis.^4^ GPA can be a devastating disorder, causing significant morbidity with a significant risk of opportunistic infection, glomerulonephritis, nasal necrosis, and upper airway obstruction.^5^, ^6^ Though mortality has declined precipitously in the past decade, recent studies still place the 10‐year survival rate as low as 40% in cases with kidney involvement.^7^, ^8^ GPA can be classified into 2 broad categories: limited or systemic, where systemic is used to indicate that renal involvement is present.^6^ It is of particular interest to the otolaryngologist because patients can present with head and neck manifestations, often as their initial presentation. Commonly involved subsites include the sinonasal cavity, ear, and larynx.^9^ Sinonasal involvement most often presents with congestion, mucosal edema and ulceration, crusts with friable underlying tissue, and epistaxis.^5^, ^10^ On examination, the septal cartilage can be involved resulting in necrosis and loss of structural integrity of the nose, leading to septal perforation or saddle nose deformity, one of the classic findings described in GPA patients.^9^ Otologic manifestations include chronic otitis media with effusion refractory to antibiotic and surgical therapy and, less commonly, facial nerve palsy and sensorineural hearing loss secondary to cochlear involvement.^11^ While subglottic stenosis, is the most common laryngeal manifestation,^8^, ^12^, ^13^ subglottic crusting and supraglottic stenosis have also been described.^14^

Approximately 80% of patients with GPA develop glomerulonephritis. This presentation is associated with a higher mortality risk,^8^ making early diagnosis essential to improving outcomes. Patients seen in the primary care or inpatient setting with any of the above complaints are generally referred for otolaryngologic evaluation. Otolaryngologists are therefore often among the first specialists to evaluate these patients. Otolaryngologists are also consulted to biopsy areas of the head and neck as this approach provides better access and a lower risk of infection than does renal biopsy. Therefore, otolaryngologists can play a key role in identifying the correct diagnosis and distinguishing GPA from other vasculitides.^7^

GPA can be a challenging diagnosis to make, particularly in patients with a limited form, due to the nonspecific nature of its symptoms. Often, the early differential is broad and includes common causes of nasal mucosal crusting such as infectious rhinosinusitis, allergic rhinitis, drug‐induced rhinosinusitis, and sarcoidosis as well as vasculitides such as microscopic polyangiitis and eosinophilic granulomatosis with polyangiitis (EGPA).^2^ Once GPA is suspected based on clinical findings, further workup generally includes a combination of serology testing (primarily c‐ANCA) and mucosal biopsy aimed at identifying small vessel necrosis in involved areas or glomerulonephritis in the case of renal biopsy. Both methods have limited sensitivity, particularly in the early course of the disease with a limited presentation. One study found that mucosal biopsy had a sensitivity of 53% and c‐ANCA testing had a sensitivity of just 47% in patients with a limited presentation of GPA^15^. The limited spread of granulomatous inflammation at this stage makes accurate biopsy difficult as c‐ANCA titers may be negative in up to 20% of all GPA patients.^15^, ^16^ As a result, there exists a subset of patients with early, limited GPA who are both seronegative and biopsy negative (double negative) upon initial presentation. To our knowledge, this is the first study to specifically address population of patients with a double‐negative presentation.

The object of this study was to describe a series of patients presenting to the Otolaryngology clinics of a tertiary care hospital with a known or suspected diagnosis of GPA. We sought to determine the percentage of patients within this population who were both seronegative and biopsy negative (double negative) at presentation and to determine the length of treatment delay in these seronegative patients.

## Methods

A database query was performed using the International Classification of Diseases‐9 diagnosis code 446.4 for all clinical sites of the Division of Otolaryngology/Head and Neck Surgery at the University of Utah to identify patients who were evaluated for known or suspected GPA from 1998 to 2021. Adults and children treated for known or suspected GPA from 1998 to 2021 were candidates for inclusion in the study. Patients for whom data on c‐ANCA status at presentation was not available were excluded from the analysis. As approved by the University of Utah Institutional Review Board, medical records were reviewed and data on presenting symptoms, extent of disease involvement, c‐ANCA status at presentation, biopsy results, time to treatment, and response to treatment were obtained. Seronegativity was defined as a c‐ANCA indirect fluorescent antibody titer of less than 1:20. Diagnosis of GPA in patient who with both negative serology and biopsy was confirmed via either later rheumatological evaluation following response to empiric treatment or later seroconversion to a positive c‐ANCA titer. Time to treatment was defined as the length in time from the receipt of results from c‐ANCA testing to the commencement of immunosuppressive therapy. For those patients who were seronegative at presentation, information on later seroconversion was obtained if possible. The documented biopsies were directed biopsies of the affected nasal mucosa. Response to treatment was defined as remission of presenting symptoms and lack of progression to renal or pulmonary manifestations (when not initially present), within 1 year of initiation of treatment, as documented in follow‐up clinical notes.

## Results

The initial database query yielded 175 patients who were evaluated for known or suspected GPA from 1998 to 2021. Thirty‐two were excluded from the analysis due to incomplete or missing data relevant to a GPA diagnosis or c‐ANCA results. Of the 143 patients who met inclusion criteria, 92 (64.3%) had GPA limited to the head and neck only, whereas 51 (35.6%) also had evidence of systemic involvement. Twenty‐six (18.2%) patients were c‐ANCA seronegative. Three (12%) of these seronegative patients had positive biopsies. Therefore, there were 23 patients with both a negative c‐ANCA titer and a negative biopsy, comprising 16.08% of the patients evaluated.

For these 23 patients with negative laboratory and biopsy results, the length of delay in treatment ranged from 0 days to 11 years. Seventeen (73.9%) of these patients presented with sinonasal disease, 12 (52.2%) presented with airway involvement, 2 (8.7%) presented with involvement of the ear, and 4 (17.4%) presented with renal involvement. Comparatively, 47 (39.1%) of patients who had positive biopsies and/or positive serology presented with renal involvement. Out of all double‐negative patients, 1 (4.3%) is known to have undergone seroconversion from c‐ANCA positive to c‐ANCA negative during the course of their disease. All patients responded to immunosuppressive therapy with either cyclophosphamide or rituximab in conjunction with corticosteroid. None of the patients with limited GPA progressed to the systemic form after they were placed on appropriate therapy (Table 1).

**Table 1:** Details of Patients Who Were Both Seronegative and Biopsy Negative at Presentation

Patient No	Presenting H&N subsite	Limited vs systemic	Time to treatment	Response (Y/N)	Seroconversion (Y/N)
5	Airway and sinus	Limited	1 mo	Y	N
7	Airway and sinus	Limited	48 mo	Y	N
15	Sinus	Limited	8 mo	Y	N
20	Sinus	Limited	0 d	Y	N
28	Airway	Limited	24 mo	Y	N
37	Airway and sinus	Limited	6 mo	Y	N
43	Sinus and ear	Systemic	2 mo	Y	Y
44	Sinus	Systemic	Unknown	Y	N
46	Sinus	Systemic	12 mo	Y	N
50	Airway and ear	Limited	21 mo	Y	N
53	Airway and sinus	Limited	60 mo	Y	N
56	Airway	Limited	Unknown	Y	N
57	Airway	Limited	11 y	Y	N
58	Airway	Limited	48 mo	Y	N
59	Airway	Systemic	0 d	Y	N
64	Airway and sinus	Limited	1 mo	Y	N
78	Sinus	Limited	12 mo	Y	N
92	Sinus	Limited	9 mo	Y	N
93	Airway and sinus	Limited	11 mo	Y	N
94	Airway	Limited	6 y	Y	N
115	Sinus	Limited	2 y	Y	N
121	Sinus	Limited	1 mo	Y	N
125	Sinus	Limited	6 mo	Y	N

Abbreviations: H&N, head and neck; N, no; Y, yes.

## Discussion

GPA is a potentially devastating illness which often initially manifests in the form of head and neck symptoms and presentations.^2^, ^17^ Once the diagnosis of GPA is suspected, the standard workup currently involves a combination of mucosal biopsy and serology testing. The most widely used serologic marker, c‐ANCA, can be invaluable in distinguishing GPA from similarly‐presenting vasculitides such as immunoglobulin G4‐related disease or EGPA.^18^, ^19^ However, we were concerned that both mucosal biopsy and serological testing may be negative in a significant portion of patients with GPA. Our aim was to determine the percentage of patients presenting to our otolaryngology clinics with known or suspected GPA who were both c‐ANCA seronegative and biopsy negative at presentation. To our knowledge, this is the first study to specifically address this subset of patients.

As suspected, our findings suggest that the prevalence of double‐negative patients is not trivial (16.08% in our cohort). This finding appears to support the preponderance of data in the literature that indicate a relatively low specificity for mucosal biopsy and c‐ANCA in the diagnosis of GPA. The literature reports a wide variation in the sensitivity of c‐ANCA testing for GPA, ranging from less than 50% to greater than 90%.^15^, ^16^ In a recent series of 82 patients with suspected GPA, the sensitivity of c‐ANCA was 47% for those with disease limited to the head and neck^20^. In patients with systemic involvement, c‐ANCA was much more sensitive (81%). The reason for this variation appears to be related, at least in part, to the fact that c‐ANCA positivity depends on disease severity and activity.^16^ Similarly, in limited cases, mucosal biopsy has been found to have a sensitivity of only 53%.^21^ Our findings would seem to support these observations as 82.6% of double‐negative patients presented with a limited manifestation of the disease, suggesting that a less severe presentation may be more prone to a negative biopsy and c‐ANCA titer.

It should also be noted that c‐ANCA has often been used in the past to monitor the progress of patient with active GPA or to monitor for relapses. Recent research has found such routine monitoring of limited value as there was no strict clinical‐immunological correspondence for up to a quarter of patients.^22^, ^23^, ^24^ These findings underscore the limitations of mucosal biopsy c‐ANCA testing and the difficulty of making the diagnosis of GPA in patients with limited disease using current diagnostic modalities. Such difficulties, in conjunction with our findings, validate the concern that current testing modalities leave a substantial number of patients prone to delayed diagnosis and suggest that diagnosis and management of GPA cannot be based on c‐ANCA and mucosal biopsy alone.

These findings should inform the most appropriate next step for the patient with a clinical presentation suggestive of GPA but with negative serology and biopsy results. If, as the data from the present series suggests, roughly 16% of patients with limited GPA will present in this manner, the diagnosis of GPA should not be ruled out based on negative serology and biopsies. Doing so will likely lead to unnecessary delays in treatment and increase the risk of further disease progression and thus morbidity and potential mortality. We propose that otolaryngologists should advocate for empiric immunosuppressive treatment of patients when GPA is highly suspected, even in the absence of a biopsy‐ or serology‐confirmed diagnosis. Our current data support this approach, given that all double‐negative patients in this series responded clinically to empiric immunosuppressive therapy as far as could be observed in the medical record.

One must, of course, weigh the risks of initiating immunosuppressive therapy against the possible benefits of preventing further disease progression. Empiric induction medical therapy for suspected limited GPA generally consists of methotrexate in combination with a corticosteroid or a corticosteroid alone. Immunosuppressants such as cyclophosphamide and rituximab are used routinely in systemic forms of GPA while methotrexate may be used in patients with cases of more mild, limited disease.^1^ It is imperative to rule out disease processes that would respond negatively to immunosuppression such as infection or malignancy. Close collaboration with and referral to rheumatologists who specialize in treatment of vasculitis is recommended.

As a retrospective review, our study is prone to limitations such as potential selection bias and an inability to determine disease incidence. Additional weaknesses stem from an inability to standardize data collection and recording procedures between providers in the electronic medical record, leading to the inconsistent use of terminology in the medical record which may have created errors in interpretation upon database query. Furthermore, at least 5 patients who were seronegative at initial presentation were known be on a regimen of immunosuppressive therapy at the time of c‐ANCA testing. Such concurrent therapy may have obscured an otherwise positive c‐ANCA titer. Response to treatment was determined by documentation in follow‐up clinic notes of either subjective improvement described by the patient or by documented evidence of improvement in physical exam or improving renal function tests in patients with a systemic presentation. Prospective studies with well‐defined and standardized criteria for treatment response would be necessary if the relationship between negative laboratory testing and treatment outcomes is to be adequately elucidated.

## Conclusions

Our series demonstrates that GPA can present without positive serologic or pathologic findings, but that the diagnosis can be made on clinical grounds. If GPA is highly suspected clinically in the setting of negative laboratory findings, we recommend empiric with immunosuppressive therapy. Furthermore, delaying treatment until c‐ANCA titers become positive is not indicated as seroconversion appears to be a rare event.

## Author Contributions

**Charles Teames**, analysis, presentation; **Julie Highland**, design, analysis; **Daniel Cox**, conduct, analysis; **Mark Elstad**, design; **Curry Koening**, design, conduct; **Marshall Smith**, design, conduct.

## Disclosures

### Competing interests

The authors report no conflicts of interest.

### Funding source

None.
